# Shoulder Pain in a Pediatric Throwing Athlete

**DOI:** 10.7759/cureus.55002

**Published:** 2024-02-26

**Authors:** Clayton R Welsh, Cassidy M Foley Davelaar

**Affiliations:** 1 Medicine, University of Central Florida, Orlando, USA; 2 Orthopedics, Nemours Children's Health System, Orlando, USA

**Keywords:** pediatric orthopedics, distal acromion, adolescent athlete, pediatric sports medicine, avulsion fracture, shoulder pain, pediatric patients

## Abstract

Pediatric avulsion fractures most commonly occur at sites of secondary ossification and are often associated with chronic stress from repetitive movements. Because of a variety of risk factors, youth athletes place higher stress on ossification centers, and their activities may predispose them to injury. This case report describes a 12-year-old female softball player who presented with pain at the distal acromion, worsened by her overhead throwing motion. Further questioning revealed improper throwing mechanics placing extenuated stress on the shoulder. Plain radiograph imaging showed an avulsion fracture of the distal acromion; conservative management with decreased mobilization and cessation of activity was recommended. Reimaging four weeks later revealed a bone-on-bone healing, and the patient was gradually allowed to return to function. This report’s discussion details the unusual location for a common injury, the mechanism of injury, an association of throwing mechanics with a shoulder injury, and recommended treatment strategies for pediatric avulsion fractures.

## Introduction

Pediatric avulsion fractures occur with both traumatic and chronic mechanisms at a variety of locations in the body. Because of the immaturity of secondary ossification centers, avulsion fractures can occur in pediatric patients with repetitive movements on account of the high strength of the muscle-bone connection as compared with the tensile strength across the physis [1,2]. Youth athletes, specifically throwing athletes, place high amounts of stress on the shoulder joint because of a variety of risk factors, including the presence of physeal cartilage, lack of surrounding muscle strength, the high number of games played per year, increased ligament laxity, and rapid long bone growth [3,4]. The case described in this report presents a distal acromion avulsion fracture in a pediatric athlete and investigates the mechanism of chronic stress and throwing mechanics that led to an avulsion fracture. The report will also detail the management and return-to-play strategy that was followed.

## Case presentation

A 12-year-old female presented to the sports medicine clinic with a complaint of right shoulder pain of three weeks’ duration. The patient was a right-hand dominant softball player and was in season at the time of the presentation. She described an acute onset of pain and swelling immediately following a right-handed throw three weeks prior. The patient described her mechanics during the causative throw as using “only her arm” with poor rotational forces generated from her body during the throwing motion. The patient described an area of discomfort at the junction between the proximal humerus and distal acromion process as well as soreness under the arm and reported pain with extension and abduction. At-home treatment with heat, ice, and over-the-counter acetaminophen and ibuprofen failed to resolve the pain. The patient reported no prior injuries to the right shoulder. She had no medical or surgical history of fracture, soft tissue injury, or orthopedic complaint, and reported taking no medications.

**Figure 1:**
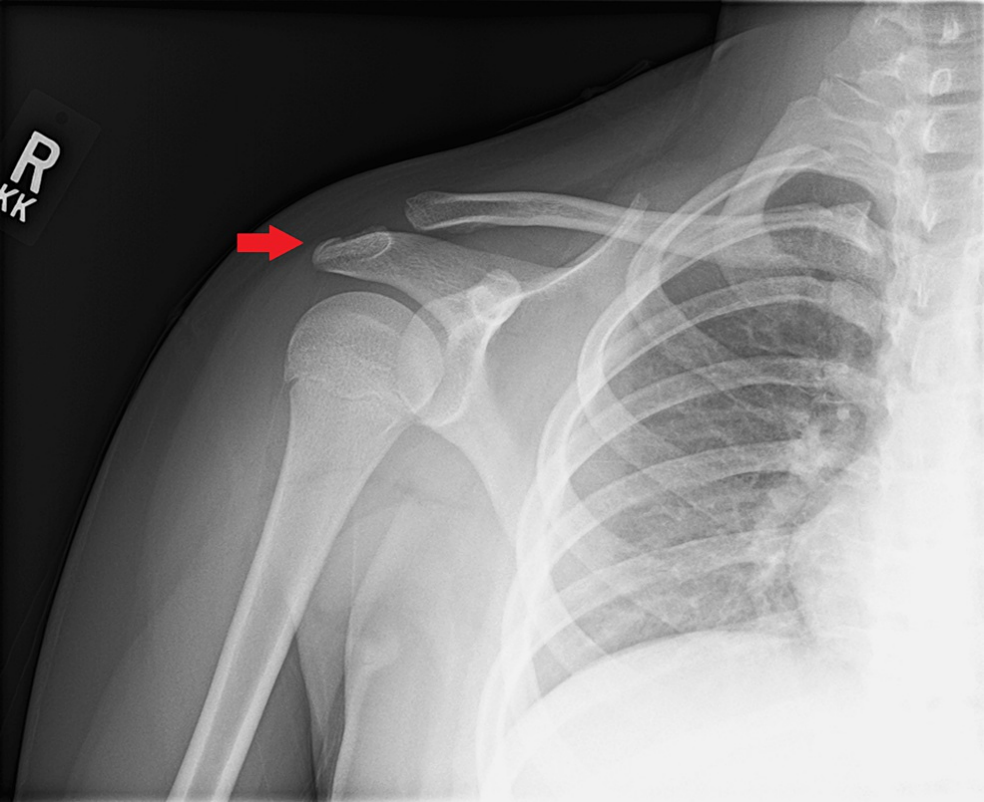
Anterior-posterior radiograph of the right shoulder in external rotation showing a non-angulated and minimally displaced avulsion fracture of the right distal acromion (red arrow)

The physical examination showed no loss of sensation over the right shoulder and an intact radial pulse. The shoulder was swollen when compared with the left but without erythema or ecchymosis, and there was no obvious deformity or Sulcus sign. The patient displayed tenderness to palpation over the supraspinatus and anterior aspect of the shoulder at the bicipital groove as well as at the distal acromion, deltoid, and proximal humerus. Passive range of motion was limited in flexion, extension, abduction, internal rotation, and external rotation with particularly limited abduction and extension. Active shoulder abduction produced significant pain. The patient displayed four out of five strength, with flexion, extension, internal and external rotation, supination, and pronation. Hawkins and Neer’s impingement tests could not be performed because of pain. O’Brien’s and Speed’s tests did not produce pain, and the apprehension and relocation test for shoulder dislocation had a negative result. There was no weakness or pain at the elbow and wrist joints. A three-view radiograph of the right shoulder taken during her visit revealed a distal acromion avulsion fracture of the right shoulder, correlating with the patient’s area of greatest discomfort (Figure 1).

The fracture showed no angulation and minimal displacement. The proximal humerus was nondisplaced, the growth plates were open, and the joint was preserved.

The patient was diagnosed with a closed, non-displaced avulsion fracture of the right distal acromion. Several diagnoses, including proximal humeral epiphysiolysis (Little League shoulder), rotator cuff pathology including superior labrum anterior-posterior (SLAP) lesions, and glenohumeral instability present with shoulder pain in the throwing athlete. Furthermore, muscle strain and shoulder ligament sprain may present with pain during the throwing motion. However, the diagnosis of avulsion fracture was apparent on plain films with separation of the bone at a site of secondary ossification as seen in Figure 1, and the patient’s most significant pain on palpation was directly over the distal acromion, thus leading to the diagnosis.

The patient was discharged from the clinic with her arm placed in a sling. She was given instructions for home exercises including flexion and extension exercises of the right elbow and pendulum circles for the right shoulder for range of motion maintenance. She was instructed to use ice with alternating heat and to avoid activities including performing a throwing motion and returning to softball.

**Figure 2:**
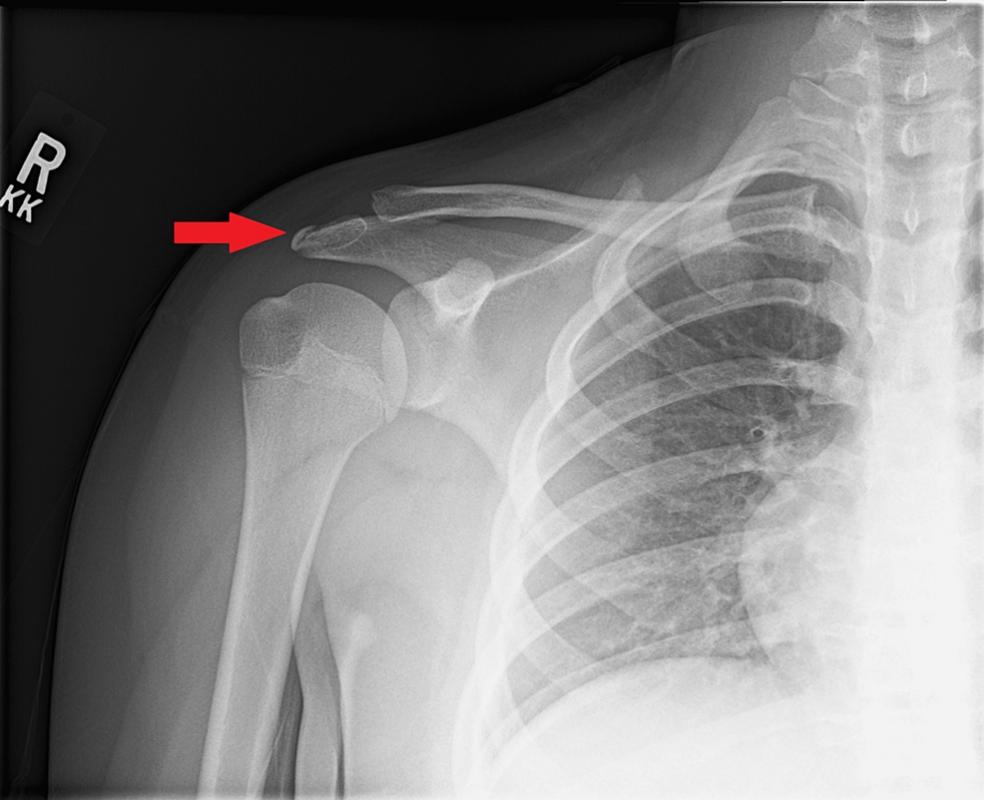
Anterior-posterior radiograph of the right shoulder in external rotation taken four weeks after initial presentation showing interval signs of healing at the distal acromion (red arrow) at the distal acromion with resolution of previous displacement and bone-on-bone healing

A follow-up visit occurred four weeks after the patient’s initial presentation. The patient reported resolved pain but stated she had not regained a full active range of motion or strength in the right shoulder. The shoulder showed decreased swelling when compared with her previous examination. At the seven-week mark, tenderness at the distal acromion had resolved and the shoulder exhibited the full range of motion with passive flexion, extension, abduction, and internal and external rotation. Strength testing revealed four out of five strength in flexion, extension, and internal and external rotation and contraction of biceps, triceps, supinator, and pronator muscles. Hawkins, Neer’s, O’Brien’s, and Speed’s tests were negative, as were apprehension and relocation. Repeated three-view radiographs of the right shoulder showed interval signs of healing at the distal acromion fracture site with no displacement and evidence of bone-on-bone healing (Figure 2).

The patient was recommended to gradually return to full activities and provided with a home exercise plan for strengthening the shoulder. A return-to-throwing protocol was encouraged, including working with a coach to improve her throwing motion.

## Discussion

Injuries to the throwing shoulder of adolescent athletes are a common occurrence among the millions of youth athletes who play baseball and softball each year with up to 32-35% of adolescent baseball and softball players reporting shoulder pain at some point during the season [3]. Identifiable risk factors for adolescent shoulder injury include weak physeal cartilage, the number of games played per year, increased soft tissue laxity, and rapid long bone development [1,2]. Additionally, throwing mechanics have become a focus of research, and improperly trained and under-conditioned adolescents are prone to breakdown of mechanics leading to increased stress on the arm [3,4]. Significant research has been conducted with regard to overuse injuries as trends show increased numbers of athletes specializing at younger ages and playing more games than in previous years [2]. Avulsion fractures occur at the bone-tendon interface and are associated with either sudden applications of force on the bone through the tendon or chronic repetitive stressing forces [5]. In adolescents, avulsion injuries are almost exclusively associated with failure through the apophysis at secondary ossification centers, as the muscle tendon fibers attaching to the bone exhibit greater strength than the calcified and noncalcified bony connection [5,6]. The pediatric acromion typically begins secondary ossification at age 10, with a fusion of primary and secondary ossification beginning at age 14 and complete by age 16 [7]. In the case described, the patient’s avulsion fracture likely occurred because of the stress associated with her throwing motion [4]. Although distal acromial avulsion fractures are not as well-described as overuse injuries, her presentation as a softball player and association with pain during her throwing motion led us to believe this may be a rare presentation of an acute-on-chronic overuse pathology in an adolescent thrower [1-3,6]. In the throwing motion, the deltoid serves to initiate arm abduction and helps slow the arm during the deceleration phase after the ball is released [8]. The medial deltoid attaches superiorly to the distal acromion, which until the average age of 18 to 25 years remains an unfused secondary ossification center [9]. In youth athletes with developing form, such as reported by the patient, improper coordination of rotational forces generated from the body with the timing of initiation of the throw and follow-through can cause aberrant stress on the bone-tendon interface and may lead to irritation, pain, and possible avulsion [8].

Because of the infrequent occurrence, management guidelines for acromion avulsion fractures are not readily available. As lower extremity avulsion fractures are more common in pediatric patients, most recommendations are tailored toward avulsion fractures of the pelvis, tibia, and calcaneus [6]. Surgical treatment is primarily aimed at correcting non-union fractures, while well-aligned avulsion fractures are often treated with limited weight-bearing and a gradual return to activity over six to eight weeks [6,10]. Specific treatment guidelines in the upper extremities include six to eight weeks of immobilization followed by physiotherapy for non-displaced avulsion fractures of the proximal humerus tuberosities [10]. In the lower extremity, common avulsion fractures of the pediatric pelvis (including anterior inferior iliac spine, ischial tuberosity, anterior superior iliac spine, iliac crest, lesser trochanter, and superior corner of the pubic symphysis) are frequently treated with conservative management including three to six weeks of partial weight-bearing followed by gradual resumption of activity [11]. Despite the frequent use of conservative management, surgical fixation of pediatric pelvic avulsion injuries has been shown to lead to higher rates of fracture union and return to sport and superior clinical outcomes as compared with conservative therapy, especially when fractures featured more than 15 mm of displacement [11]. While similarly presenting conditions, such as muscle strain and apophysitis benefit from an early range of motion exercises, avulsion injury can be further displaced and worsened without limitation of movement. Rest across the apophysis assists in reunification of the fibrous connection and a decrease in pain, thus leading to the recommendation for conservative management. In our case, reassessment of our patient after four weeks of immobilization showed healing without signs of nonunion, and the patient was able to begin a return-to-throwing protocol.

## Conclusions

An avulsion fracture of the distal acromion is a rare occurrence that has not been well-described in the literature. Pediatric patients are at a higher risk for avulsion fractures associated with chronic injury because of the presence of secondary ossification centers with developing bone. In this case report, a 12-year-old softball player with a distal acromion fracture illustrates the successful management strategy of six to eight weeks of limited mobilization with a gradual return to play for avulsion fractures featuring minimal displacement. Improper throwing mechanics, as featured in this patient, increase the risk of joint injury, and proper form should be emphasized in all youth athletes with careful consideration placed toward preventing overuse injury.
